# Diversity of dsDNA Viruses in a South African Hot Spring Assessed by Metagenomics and Microscopy

**DOI:** 10.3390/v9110348

**Published:** 2017-11-18

**Authors:** Olivier Zablocki, Leonardo Joaquim van Zyl, Bronwyn Kirby, Marla Trindade

**Affiliations:** Institute for Microbial Biotechnology and Metagenomics, Department of Biotechnology, University of the Western Cape, 7535 Bellville, South Africa; olivier.zablo@gmail.com (O.Z.); vanzyllj@gmail.com (L.J.v.Z.); bkirby@uwc.ac.za (B.K.)

**Keywords:** hot spring, metavirome, *Fuselloviridae*, jumbo phage, archaeal viruses, freshwater cyanophages, *Gemmata*

## Abstract

The current view of virus diversity in terrestrial hot springs is limited to a few sampling sites. To expand our current understanding of hot spring viral community diversity, this study aimed to investigate the first African hot spring (Brandvlei hot spring; 60 °C, pH 5.7) by means of electron microscopy and sequencing of the virus fraction. Microscopy analysis revealed a mixture of regular- and ‘jumbo’-sized tailed morphotypes (*Caudovirales*), lemon-shaped virions (*Fuselloviridae-*like; salterprovirus-like) and pleiomorphic virus-like particles. Metavirome analysis corroborated the presence of His1-like viruses and has expanded the current clade of salterproviruses using a polymerase B gene phylogeny. The most represented viral contig was to a cyanophage genome fragment, which may underline basic ecosystem functioning provided by these viruses. Furthermore, a putative *Gemmata*-related phage was assembled with high coverage, a previously undocumented phage-host association. This study demonstrated that a moderately thermophilic spring environment contained a highly novel pool of viruses and should encourage future characterization of a wider temperature range of hot springs throughout the world.

## 1. Introduction

Terrestrial hot springs are generally considered low complexity environments, composed of a few key microbial taxa [1]. Due to constant high temperatures, often associated with high acidity, hot springs have been shown to harbor unique consortia of thermophilic bacteria and archaea [2]. Hot spring virus communities, while having received attention in recent years, have been limited to only a few thermal springs, mainly sampled from Yellowstone National Park in the United States [3,4,5,6,7]. The majority of these reported virus community analyses were conducted in boiling, acidic springs (between ca. 70 and 92 °C, pH 1.8–5.5), shown to be dominated by archaeal viruses of the *Rudiviridae*, *Lipothrixviridae* and *Ampullaviridae* families. Currently, the number of full-length, sequenced archaeal virus genomes remains small (78 as of February 2017), reflecting the limited number of studies that have focused on the characterization of archaeal viruses. Therefore, the metagenomic analysis of novel hot springs brings two main benefits: (1) expand upon the general microbial diversity of hot springs throughout the world and (2) permit the addition of novel virus genomes to databases, including (but not limited to) archaeal viruses.

In South Africa, over 80 hot springs have been documented, with a select few solely used for recreational purposes, while others are used for religious practices and places of worship [8]. To date, microbial analysis of South African hot springs has been limited to those in the Limpopo province and only assessed the bacterial 16S ribosomal RNA (rRNA) diversity [9,10,11], while no virus community analysis has been conducted in any of the hot springs. However, two publicly available shotgun metagenome datasets generated from two South African hot springs—Sagole and Tshipise—have recently been annotated by the Joint Genome Institute (JGI) Integrated Microbial Genomes/Virus (IMG/VR) platform. One of the hottest recorded hot springs in South Africa, Brandvlei, is classified as a scalding spring [12]. Due to its near neutral pH (5.7), low salinity (73.5 µS/cm) and moderately high temperatures (60 °C), this spring may be considered as an ‘intermediate zone’, in comparison to mesophilic springs and true high temperature (i.e., boiling) thermal springs. This provided the opportunity to investigate the virus community associated with an African scalding spring, which would expand the number of viromics analyses conducted in the limited range of terrestrial hot springs. In this study, the virus community analysis was conducted using electron microscopy and metagenomics to assess the viral content of the spring.

## 2. Materials and Methods

### 2.1. Sample Collection and Virus Enrichment

Water was collected from the Brandvlei hot spring (BHS), located in the Western Cape, South Africa (GPS coordinates: −33.732496, 19.413317). Temperature and pH were recorded in triplicate readings (Cat. No. 2502, Crison pH-meter 25, Alella, Spain,). Approximately 720 L of spring water were pumped at the water source (Masterflex I/P Peristaltic Pump, Merck; Model No. XX80EL000, Darmstadt, Germany) and concentrated against a 30 kDa cutoff prep scale tangential flow filtration (TFF) cartridge (Millipore Corp., Bellerica, MA, USA; Cat. No. CDUF006LT) to a final retentate volume of ≈300 mL. The TFF concentrate was kept at room temperature until processing. Water sediments and eukaryotic cells were removed by low speed centrifugation (3000× *g*) for 10 min. Bacterial cells were removed by filtering the supernatant through a 0.20 µm filter (Whatman Puradisc FP 30 CA-S Syringe Filter, Cat. No. 09302152, Maidstone, United Kingdom). Viruses were further concentrated through Amicon 50 kDa molecular weight cutoff (MWCO) ultra-centrifugal filters (Millipore Cat. No. UFC905024, Burlington, Massachusetts, USA), until a 1500 µL final volume was reached. An additional round of centrifugation was performed to exchange the original BHS water with a 1× physiologically-buffered saline (PBS; pH 8) solution.

### 2.2. Electron Microscopy

A 200-µL fraction of the concentrated water sample was prepared for transmission electron microscopy (TEM) according to Ackerman (2009) [13]. Briefly, the virus concentrate was centrifuged for 1 h at 25,000× *g*. The supernatant was decanted, and the viral pellet was resuspended in 0.1 M ammonium acetate solution (pH 7.0). The process was repeated twice, and the pellet was resuspended in a final volume of 10 µL. Virus particles were stained with 2% uranyl acetate and visualized with a Tecnai 20 electron microscope operating at 200 kV (University of Cape Town, Cape Town, South Africa).

### 2.3. Virome DNA Purification, Sequencing and Read Assembly

Viral concentrates were treated with 1 U of DNase I (Cat. No. EN0521, ThermoFisher Scientific, Waltham, Massachusetts, USA) and incubated at 37 °C for 60 min. The presence of free, non-viral DNA was checked by the amplification of the 16S rRNA gene (primers E9F [14] and U1510R [15]). The reaction mixture for PCR consisted of: 1.5 µL of metagenomic DNA, 2.5 µL of each primer (10 mM), 2.5 µL of 2 µM deoxynucleoside triphosphates (dNTPs), 2.5 µL of 10× DreamTaq buffer (ThermoFisher Scientific), 1 µL of 10 mg/mL bovine serum albumin (BSA), 0.125 µL DreamTaq polymerase (ThermoFisher Scientific) and Milli-Q water to a final volume of 25 µL. The concentrate was treated with 50 µg/mL Proteinase K (Cat# EO0491, ThermoFisher Scientific) and incubated for 2 h at 55 °C. Twenty percent sodium dodecyl sulfate (SDS) was added, incubated at 37 °C for 60 min. DNA was extracted by performing three rounds of phenol extraction and two rounds of chloroform extraction. DNA was precipitated by adding a 1/10 volume of 3M sodium acetate (pH 5.0) and a 2.5× volume of 100% ethanol and incubated overnight at −20 °C. The DNA was pelleted by centrifugation at 14,000× g for 60 min. The pellet was washed with 70% ethanol and sequentially resuspended in 30 µL Tris-HCl buffer (10 mM). DNA was further cleaned using the Qiagen MinElute reaction clean-up kit (Cat. No. 28204, Qiagen, Hilden, Germany) as per the manufacturer’s instructions. This was followed by DNA fragment size selection using the NucleoTrap kit (Cat. No. 740584, Macherey-Nagel, Düren, Germany). Size-selected sequencing libraries were prepared using the Nextera XT kit (Cat. No. FC-131-1024, Illumina, San Diego, CA, USA) and sequenced (2× 250 bp) using MiSeq Reagent Kit v3 chemistry (Cat. No. MS-102-3003, Illumina). Sequencing was performed at the Institute for Microbial Biotechnology and Metagenomics (IMBM, University of the Western Cape, Bellville, South Africa). Reads were imported as paired-end in CLC Genomics Workbench 7.5 (CLC bio, Aarhus, Denmark). The dataset was curated using default parameters in CLC for trimming based on quality (limit = 0.05) and number of ambiguous base removals. Reads were mapped (length and similarity fraction of 95%) against the human genome assembly GRCh37 (obtained from https://www.ncbi.nlm.nih.gov/grc/human/data?asm=GRCh37) set in order to remove potential contamination. Curated reads were assembled (within CLC Genomics) into contigs, using parameters of (length fraction = 0.8 and similarity fraction 0.8). Reads were uploaded to EBI Metagenomics (https://www.ebi.ac.uk/metagenomics/) under Project ID ERP020382. VirSorter [16] was used for binning (and annotation) of viral contigs from the assembled sequence dataset. Two predicted, putative phage genome fragments, *Gemmata* phage and cyanophage BHS, were individually submitted to the GenBank database under Accession Numbers MF098554 and MF098555, respectively. Contigs were also submitted for additional gene annotation to the online analysis platform Integrated Microbial Genomes (IMG, available at http://jgi.doe.gov/) with Genome Online Database (GOLD) Project ID Gp0177339. The sequencing run statistics, quality control metrics and diversity indexes are summarized in Table S1.

### 2.4. Viral Diversity Predictions

Viral richness and evenness were predicted by first generating a contig spectrum through CIRCONSPECT [17] with an assumed average phage genome length of 50 kb [3]. The contig spectra were then used to determine the best fitted model (i.e., lowest error) in the Phage Communities from Contig Spectrum (PHACCS) package [18]. For the Brandvlei dataset, the best fitted model was logarithmic, as it yielded the lowest error.

### 2.5. Phylogenetic Analyses of *terL* and *polB2* Genes

The large terminase (*terL*) gene phylogram was constructed using all available TerL protein sequences retrieved from the IMG/VR database [19], as well as TerL sequences identified in other hot spring metagenomic datasets, including South African springs [9]. A total of 660 reference phage TerL (from virus isolates) and 471 hot spring-derived TerL sequences (derived from Brandvlei, Great Boiling Spring, Tshipise, Sagole, Octopus and Nymph Lake) constituted the final dataset. The terminase domain was aligned at the amino acid level with the online version of MAFFT [20], and a tree was constructed using the weighted pair group method with arithmetic mean (UPGMA) method for sequence-based similarity hierarchical clustering. This method allowed a greater number of sequences to be compared together and allowed for high variability in the input sequence set. Visualization and tree analysis was generated with iTOL [21].

Analysis of the family B DNA polymerase protein sequences (PolB) was conducted by retrieving IMG database PolB sequences using keyword pfam03175, denoting the virus-related domain of the *polB* gene. The queried database results were filtered to only include archaeal viruses and phages, which totaled 26 sequences. These reference sequences were pooled with all PolB sequences from the BHS virome dataset. The amino acid PolB sequences were aligned with MUSCLE v3.8.31 [22] using the highest accuracy setting within the phylogeny.fr (http://www.phylogeny.fr/; [23]) online pipeline. Phylogenetic reconstruction was performed using the maximum likelihood method [24] implemented in the PhyML program (v3.1). The WAG substitution model was used, and the gamma shape parameter was estimated directly from the data. Reliability for the internal branch was assessed using the approximate likelihood ratio (aLRT) test [25]. Visualization was generated with iTOL.

### 2.6. CRISPR Predictions and Host Assignments

The CRISPR loci were searched in the curated read dataset, using Minced (https://sourceforge.net/projects/minced/), a metagenomic version of the CRISPR recognition tool (CRT) source code [26], using the ‘minimum number of repeats to find” (minNR) to 3 and the rest set to defaults. To assign Brandvlei spacer regions to database isolates, the BHS CRISPR dataset was searched using nucleotide BLAST against the CRISPR webserver database (available on the CRISPR webserver at http://crispr.i2bc.paris-saclay.fr/; [27,28]). Unique (i.e., non-redundant) spacer sequences (20 in total) were identified with significant homology to database isolates bearing the same spacer.

### 2.7. PCR Amplification, Cloning and Sequencing of the Gemmata-Related Terminase Gene

The *terL* gene identified in the putative *Gemmata* phage contig was amplified using a single primer pair (5′-3′; forward: GTCGGTACTGGGAGTAGGGT; reverse: ATCGAGCCAGTTAGGGGACT). A 25 μL PCR reaction consisted of 10 mM of each primer, 2 μm dNTPs, 10× DreamTaq buffer (ThermoFisher Scientific, Cat. No. B65), 10 mg/mL bovine serum albumin (Merck, Cat. No 810037) and 1 U DreamTaq polymerase (ThermoFisher Scientific, Cat. No. EP0701) suspended in Milli-Q water. Thermal cycling was conducted in a Bio-Rad T100 (Cat. No. 1861096) under the following conditions: 95 °C for 5 min, 34 cycles of 95 °C for 30 s, 58.7 °C for 30 s, 72 °C for 60 s and final extension of 10 min at 72 °C. Amplicons of expected size (690 bp) were cloned into pGEM-T Easy Vector (Promega, Cat. No. A1360) and Sanger sequenced at the Stellenbosch University Central Analytical Facilities, Stellenbosch, South Africa.

## 3. Results

### 3.1. General Characteristics of Brandvlei Hot Spring

Brandvlei hot spring is the hottest, naturally occurring terrestrial geothermal spring in South Africa (Figure S1). The recorded, on-site pH and temperature were 5.7 and 60 °C, respectively, in accordance with previous survey measurements of this site [29,30]. Thus, this spring can be considered mildly acidic and thermophilic (i.e., scalding). Moreover, it has been found to contain trace elements such as lithium and nickel and has been reported as radioactive due to the detection of moderate radon quantities ranging from 673 to 713 Becquerel/liter (Bq/L) [12,31]. The water was almost completely transparent. Around the contours of the spring, green microbial mats-patches were found stretching 30–50 cm towards the spring epicenter.

### 3.2. Electron Microscopy

**Figure 1:**
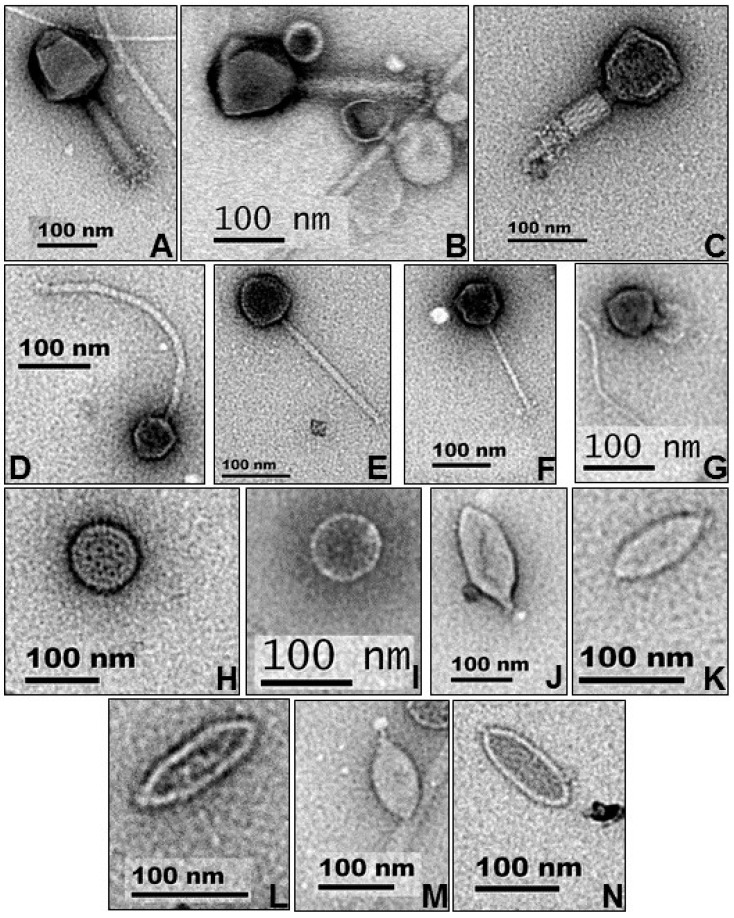
Morphological diversity of observed virus-like particles in Brandvlei hot spring. Each bar represents 100 nm. (**A**–**C**) *Myoviridae*; (**D**–**F**) *Siphoviridae*; (**G**) *Podoviridae*; (**H**–**I**) Unclassified; (**J**–**N**) *Fuselloviridae*-like.

A subset of representative virus morphotypes is shown in Figure 1, selected from a total of 74 observed virus-like particles (VLPs) (Table 1). Most of the identified VLPs were tailed bacteriophages (order: *Caudovirales*); however, this may be an overestimation of this group of viruses as some archaeal viruses are known to have a tailed morphology [32]. The lambda-like siphoviruses were dominant (average head diameter of 69 nm), with long (range: 58–318 nm), non-contractile tails (Figure 1D–F). Of the observed myoviruses (Figure 1A–C), two similar-sized ‘jumbo’ phage particles were present [33,34], with identical head diameters of 129 nm and thick, contractile tails averaging 166 nm in length. Lemon-shaped VLPs (Figure 1J–N), putatively assigned here as probable halophilic/thermophilic archaeal virus groups, which include the genus *Salterprovirus* and members of the *Fuselloviridae* (e.g., SSV1*e*) [35,36,37,38] were also visualized. This distribution of morphotypes suggested a heterogeneous virus community composed of both bacteriophages and archaeal viruses in the hot spring sample.

### 3.3. Analysis of the Most Represented Viral Genome Fragments

From the 9220 assembled contigs, 84 were predicted by VirSorter as being viral in origin, inclusive of all probabilistic categories (I, II and III) according to VirSorter. Only contigs of ≥5 kb in length were retained after this selection, which left 15 viral putative partial/full virus genomes in the nucleotide size range of 5387–26950 nt and read coverages ranging from 7×–192×). This set of contigs was manually inspected for gene affiliation through BLASTx analysis against the National Center for Biotechnology Information (NCBI) non-redundant (nr) database in order to further re-assess each contig’s validity as a genuine virus genome fragment. In this section, the two most represented contigs (in terms of read coverage) are presented in more detail.

The contig with the most read coverage (192×) in the sample was a 10 kb fragment with nine predicted open reading frames (ORFs) (cyanophage BHS, Accession Number MF098555). Six of these were attributed to phage functions, including a terminase, major capsid protein, two tail tube (A and B) proteins and a portal protein. Most of these identified phage proteins had very high similarity BLASTx hits to cyanophage PP (Accession Number NC_022751) and *Phormidium* phage Pf-WMP3 (Accession Number NC_009551), both closely-related podoviruses [39,40]. Whole genome comparisons between these genomes showed near identical gene organization (Figure S2), with high sequence similarity at the amino acid level. Both cyanophage genomes were compared at the amino acid level (tBLASTx) to the BHS contig, which showed almost identical gene synteny and high sequence similarity toward the 3’ half of both reference cyanophages. Despite additional reference read mapping to these cyanophages and local BLAST searches using the contig dataset, the 5’ half of the two reference genomes could not be mapped. To rule out this contig as being microbial contamination in origin, read mapping at high similarity stringency (95%) to the two reference cyanophage hosts (*Leptolyngbya boryana* and *Phormidium tenue*) yielded a very low and sporadic number of read matches (188–362) across these host genomes. This indicated that it is less likely that this contig resulted from cyanobacterial genomic DNA contamination and that this contig represented a genuine cyanophage genome fragment, delimited at gene boundaries.

The second most represented viral fragment (*Gemmata* phage BHS, Accession Number MF098554; 52× coverage) in the assembled dataset was 26950 bp in size, with 30 predicted ORFs. Most (63%) predicted peptides had no functional homologs in the NCBI nr database, using BLASTx searches. Eleven phage-related genes were identified, including tape measure protein, major capsid protein E, methyl transferase and large/small terminase subunit domains. This gene set inferred a lambda-like, temperate siphovirus-like genome composition. The genome also encoded a conserved DNA polymerase I domain, with 3′-5′ exonuclease activity (COG0749). Interestingly, higher classification of this polymerase showed significant similarity to the *Aquificae*-like domain (cd08639), typically found in strictly thermophilic, Gram-negative rods [41]. Most of the other genes with hypothetical functions on this contig were taxonomically affiliated with the eubacterial, *Gemmata* sp. SH-PL17 (Accession CP011271.1), a member of the phylum Planctomycetes. In particular, the large terminase gene had a BLAST score of 231 with 31% similarity to two *Gemmata* isolates (both top BLASTx hits), bearing the terminase conserved protein domain. This hinted at undescribed/misannotated (pro)phage genes within these *Gemmata* genomes. Currently, no isolated *Gemmata* phage genomes are available for comparison. The identified BHS *Gemmata* phage-like terminase gene was successfully cloned and amplified (690 bp fragment) from the initial metavirome DNA extract, raw hot spring water and algal supernatant from the spring. These samples were collected six months after initial sampling and contained a strong positive signal in every sample tested. This not only adds confidence to the sequence assembly, but also suggested that this putative phage is a persistent (i.e., actively replicating) member of the microbial community in this hot spring.

### 3.4. Diversity of Bacteriophages

Taxonomic classification of predicted virus genes against the IMG/VR database (25) showed a dominance of all three tailed phage families (*Caudovirales*), as is often observed in metavirome datasets (Table 2). At the virus isolate level, a large fraction of genes was related to cyanophages (e.g., cyanophage PP, *Phormidium* phage) from the *Siphoviridae* and *Podoviridae* families. Furthermore, the contig with highest coverage in the assembled dataset consisted of a cyanophage genome fragment (Figure S2). The abundance of these phages may be related to the extensive growth of autotrophic microbial mats around the contours of the hot spring, which may sustain a release of these phages in the surrounding water column.

A second category of well-represented phages were ‘jumbo phage’ isolates, particularly *Bacillus* virus G (*Myoviridae* family, Accession NC_023719 [42]). In total, 101 predicted genes were mapped to various genomic regions of 23 currently recognized jumbo phage reference isolates [34], 38% of which were to *Bacillus* phage G. Gene functions related to this phage identified in the BHS dataset included DNA polymerase family A, baseplate assembly protein and tail sheath proteins. However, no contigs or partial genome assembly related to the virus group were identified, thus limiting genetic characterization of these phage in the BHS sample. Nonetheless, compounded by microscopy observations, this strongly suggests the presence of these viruses in this ecosystem.

A large number of lysis-related (e.g., peptidases) genes were identified (147), several of which were present on predicted virus genomes. Hallmark genes belonging to tailed phages were searched for in the assembled dataset and included integrases (41 genes), terminases (53 genes), portal proteins (32 genes), holin superfamily 3/6 (6 genes), tail-related (65 genes) and capsid proteins (29 genes). The identified capsid proteins were functionally homologous to major capsid protein (MCP) gp23 (Enterobacteriophage T4; *Myoviridae*: *Tevenvirinae*), MCP E (Enterobacteria phage lambda; *Siphoviridae*: Lambdavirus) and phage phi-C31 MCP (*Streptomyces* phage phiC31; *Siphoviridae*: Phic31virus).

The terL subunit (a hallmark marker for the *Caudovirales* [43]) was used to provide further phylogenetic resolution in terms of bacteriophage diversity within the hot spring dataset. A global phylogram (Figure S3) was created, which included 53 BHS *terL* sequences, hot spring sequences (Octopus (USA), Sagole (South Africa) and Tshipise (South Africa)) and RefSeq phage isolates. The average amino acid length of aligned terL protein sequences was 515. BHS terminases were spread throughput the tree, which included both mesophilic and thermophilic phages. There were no unique BHS clades, but most BHS terminases were clustered with Sagole and Tshipise sequences, which formed three major hot spring clusters. Several other BHS clusters were related to *Arthrobacter* phage BarretLemon, *Haloarcula californiae* head-tail virus (HCTV)-1, *Halorubrum* sp. s5a-3 Halovirus HRTV-4, Bacteriophage Mu and *Shewanella* phage 1/44. Some closely related phage isolates to Brandlvei isolates included *Streptococcus thermophilus* phage DT1/Sfi19, *Xanthomonas* phage CP1, *Thermus* phage P23-43, *Sulfitobacter* phage NYA-2014a, *Synechococcus* phage S-CAM8 and several *Pseudomonas* and *Mycobacterium* phages.

### 3.5. Diversity of Archaeal Viruses

#### 3.5.1. Identification Using the *polB2* Gene

**Figure 2:**
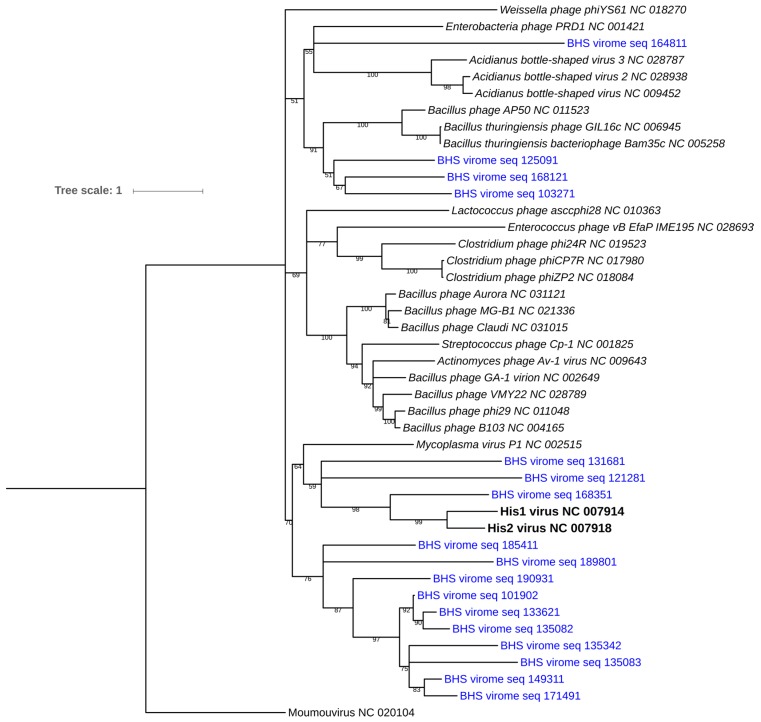
Maximum-likelihood phylogram of the viral polymerase B protein domain. Bootstrap confidence values are expressed in % and are indicated along the branch lengths. Branches with support values lower than 50% were collapsed. Phylogenetic distance is denoted by branch length (to scale) and is indicated in the legend. The Moumou virus (an algal virus) was placed at the bottom of the tree as an outgroup. PolB sequences from the BHS dataset are highlighted in blue and denoted as “BHS virome seq (sequence ID)’. His1 and His2 PolB sequences are shown in bold. All NCBI accession numbers for the reference isolate sequences are indicated after the strain denomination.

Genes or genome sequences belonging to archaeal viruses in the IMG/VR database could not be matched to any predicted metavirome genes. However, this most probably resulted from the very low number of archaeal viruses genomes currently deposited in the database (<80 isolate genomes). Furthermore, archaeal virus genomes most often contain genes with no homology to other virus groups, as well as very little DNA similarity to other archaeal virus families [44]. However, DNA polymerase family B domain (pfam03175) has been found to be shared across several archaeal virus genomes [32]. In the Brandvlei dataset, 17 PolB domain sequences were identified and aligned to a reference set extracted from all RefSeq virus genomes containing this gene. Expectedly, very few hits were found and included His1/His2 [45] and *Acidianus* bottle-shaped virus (ABV) [46]. In total, 43 sequences were included in the phylogenetic analysis with an average sequences length of 516 amino acids. Figure 2 shows the PolB phylogram, with Moumou virus (an algal virus [47]) as the outgroup. Sequence clustering showed three Brandvlei PolB sequences closely related to His1/His2 viruses, expanding the salterprovirus-like clade. His1/2 viruses have only been associated with archaea and are the sole isolates representatives of the *Salterprovirus* genus [48]. The presence of the virus group is further reinforced by microscopy (Figure 1J–N), where short-tailed type [45] lemon-shaped VLP morphologies were observed. The remaining majority of Brandvlei PolB sequences clustered into a single clade, with no direct relationship to reference sequences. A single BHS PolB sequence was present within the *Acidianus* bottle-shaped virus1-3 cluster, isolated from a boiling, acidic spring [46]. Three Brandvlei PolB2 sequences appear to be of bacteriophage origin given the close association with *Bacillus* phage AP50 and two *Bacillus thuringiensis* phages.

#### 3.5.2. Identification of Archaeal Viruses Using Reference Genome Read Mapping

The metavirome was searched for the presence of archaeal viruses through mapping of the metavirome reads to a set of reference archaeal virus genomes. The reads were mapped at various stringency parameters (length fraction: minimum sequence length that must match the query; and similarity fraction: minimum percentage similarity match against the query) to a total of 78 full-length archaeal virus genomes retrieved from the NCBI RefSeq database. The best genome hits at the various parameters are shown in Table S3. Almost consistently across most mapping stringency scenarios, HCTV was the best hit. Two other isolates, *Sulfolobus tengchongensis* spindle-shaped virus 2 (STSV2) and *Sulfolobus islandicus* rudivirus 3, were also identified at 80% similarity along 60–80% of the length fraction. HCTV has a siphovirus morphology (i.e., head-tail virions, with long, non-contractile tails) [49]. As the vast majority of the observed virions in the microscopy analysis were *Siphoviridae*-like, it is thus a possibility that some are in fact archaeal viruses and not bacteriophages. Given the thermophilic nature of the Brandvlei spring, this is a reasonable conclusion. Furthermore, three *Haloarcula* isolates (see Table S2) have been identified through the IMG pipeline, lending further support for the presence of HCTV-like viruses in the hot spring sample.

#### 3.5.3. Identification of Archaeal Virus Genes by Using Host Functional Gene Annotations

Virus genes associated with archaea (and bacteria) are often taxonomically annotated as host genes, thereby underestimating the number of virus sequence hits within automated metagenomic pipelines used to characterize metavirome datasets [50]. To maximize the recovery of archaeal virus genes in the BHS virome, putative archaeal virus-related genes were identified by searching IMG/VR-identified archaeal genomic hits based on protein domain functions of the predicted ORFs. In the BHS virome, 31 predicted ORFs were initially taxonomically associated by the IMG pipeline with several members belonging to Archaeal phyla (Crenarchaeota, Euryarchaeota and Thaumarchaeota). Upon manual examination, the functional annotations (cluster of orthologuous groups (COG) and protein family database (PFAM) categories) of most genes were related to phage-encoded functions (Table S2). For example, these genes included phage terminases (virus assembly), baseplate (virus assembly), peptidases (host lysis), DNA methylases (host defense evasion) and a CRISPR locus (host evasion). While some identified genes could possibly be genuinely host-associated, five out of the 31 ORFs were definite phage-encoded functions. The archaeal isolates with the most probable virus genes included *Archaeoglobus sulfaticallidus* strains PM70-1 and DSM 19444, *Methanobrevibacter curvatus* strain DSM11111, *Methanolobus psychrophilus* strain R15 and *Methanomethylovorans hollandica* strain DSM 15978. None of these archaeons have any reported phage/prophages, and the gene similarity most probably represents related species to those in the Brandvlei hot spring. These findings have further highlighted the hidden archaeal virus diversity within databases.

### 3.6. CRISPR-Guided Virus-Host Associations

The CRISPR loci, widely spread amongst bacterial and archaeal genomes, forms part of the CRISPR-Cas system, which serves as a virus evasion mechanism [51]. Within the CRISPR array, spacer sequences are found between direct repeats (DR), derived from virus segments (protospacers) incorporated into the array following a successful virus evasion. Thus, spacer sequences found within genomes may serve as a ‘record’ of past infections [51] and have been used to infer virus-host pairs [6,52]. In the BHS virome, 67 CRISPR sequences were predicted, of which 14 unique (i.e., to a single isolate) had significant homology to the CRISPR database. About half of the identified isolates (Table 3) were either thermophiles or hyperthermophiles and included members of the Euryarchaeota (e.g., *Thermococcus*), Crenarchaeota (e.g., *Pyrobaculum*) and Proteobacteria (e.g., *Pseudomonas*, *Myxococcus*). These results suggest a heterogeneous community of bacteria and archaea in the hot spring.

## 4. Discussion

This study has provided the first exploratory virus community investigation of an African hot spring, located in the Western Cape, South Africa. Electron microscopy analysis and metavirome data were used to determine the virus community composition of the hot spring, which appears to have a mixed community of both bacteriophages and novel archaeal viruses. It should be noted that the sequencing chemistry used (i.e., Nextera) would selectively enrich for dsDNA molecules and that BLAST-based searches are limited by the availability of virus sequences in public databases.

The heterogeneous virus population, as revealed by the terminase phylogeny, most probably resulted from the moderately thermophilic hot spring temperature constraints imposed by the hot spring ecosystem, which would allow for both (mildly) thermophilic bacteria and archaea to thrive [53]. The diversity of CRISPR loci, predicted and identified in the metavirome data, has provided further indications regarding the prevalence of both thermophilic bacteria and archaeal genera as putative viral hosts in the Brandvlei hot spring. Furthermore, the diversity of phages and archaeal viruses observed in Brandvlei hot spring is in stark contrast with previously studied hot springs, typically dominated by archaeal virus members such as *Ampullaviridae* and *Lipothrixviridae* [6], none of which were identified by read mapping and contig affiliations in this study. The divergence in terms of virus diversity is most probably temperature- and pH-driven, as these two variables lie in more extreme values in other boiling spring viromics analyses. Our results have shown that a mildly thermophilic hot spring harbors a more complex virus population than previously assumed, and future metagenomic studies of additional hot springs of this temperature range should be carried out to further consolidate this hypothesis. Cyanophages appear to be the dominant viruses in this hot spring. Genomic analysis of the cyanophage BHS contig showed high similarity in genomic synteny and amino acid sequence identity within this group of viruses, despite high divergence in environmental conditions (i.e., ocean versus scalding freshwater). Little is known with regards to the ecology, diversity and distribution of (thermophilic) freshwater cyanophages [54,55], despite their key ecological roles in other aquatic environments such as nutrient cycling in the marine environment [56,57]. Worth noting is that in Siloam hot spring (58 °C), located in a different province of South Africa, cyanobacteria were reported as one of the dominant phyla within the microbial community using a 16S rRNA-based metagenomic approach [58]. Future research should aim at the isolation and characterization of freshwater cyanophages in other hot springs. In addition, temporal and quantitative analysis with regards to virus-host ratios (with an emphasis on phototrophic cyanobacteria) would provide novel insights into microbial population dynamics within hot springs.

The assembly of a 26 kb, partial *Gemmata*-predicted virus genome is a novel addition to the currently recognized range of phage hosts. Similar to cyanobacterial abundances, *Gemmata*-like species have also been reported to be among the most abundant species in the Siloam hot spring [58]. It is thus probable that thermophilic *Gemmata*-like Planctomycetes members are present in BHS, which are yet to be isolated and characterized. There are currently no reports of isolated *Gemmata* phages; however, an incomplete prophage-like region has recently been identified in the mesophilic *Gemmata massiliana* genome [59]. The archaeal virus diversity consisted of several relatives of RefSeq type isolates, such as His1/2 and HCTV. These viruses and cognate hosts are known thermophiles and/or halophiles [48,60]. While His1/2-like lemon-shaped particles were observed in this study, HCTV virions are tailed, *Siphoviridae*-like [60]. Furthermore, haloarchaeal virus morphotypes span all tailed virus families, as well as pleiomorphic morphotypes [61]. Thus, there remains uncertainty whether the majority of the observed virions are bacteriophages or constitute an archaeal virus population. Given the positive identification of several novel, closely-related His1-like and various halophilic archaea (e.g., HCTV) at the genetic level in the Brandvlei sample, we suggest that members of this sequence cluster may not be solely associated with halophiles, but rather mildly thermophilic host strains within this group of viruses. Further isolation and characterization of these isolates will allow clarification of this hypothesis.

There is currently a limited number of characterized bacteriophages that possess genomes of a size ≥200 kb, termed ‘jumbo’ phages [33]. To date, the majority of jumbo myoviruses have been isolated from water samples [34] and infectious towards members of the *Enterobacteriaceae* (e.g., *Cronobacter* phage GAP32, *Klebsiella* phage Rak2) and *Bacillus megaterium* (*Bacillus* phage G). In the BHS sample, both microscopy and single gene affiliations supported the presence of jumbo myoviruses. We could not, probably due to the lack of sufficient DNA and no random amplification prior to sequencing, assemble a full/partial genome. However, future studies will aim to enrich for this elusive jumbo phage. To our knowledge, this is the first observation of environmental, potentially thermotolerant jumbo myophages and expands the known range of environmental conditions from which these viruses can be isolated. The identification of CRISPR regions and phage-specific functions belonging to thermophiles hinted at an unexplored virus-host diversity, with a high potential for the discovery of novel thermophile-associated viruses. Despite a certain database bias involved in our results (i.e., low number of reference virus genomes), there was a high number of thermophiles with currently no recognized associated viruses that were identified. Functional annotations revealed that phage proteins were present in reference Archaeal genomes, and homologs of these genes were identified in the sample. This suggested that these reference isolates harbored prophage regions, and our study complements this assertion given that our method would select for the recovery of extracellular, encapsidated particles. This may encourage future studies involving archaea to perform lytic induction experiments, which would greatly aid virus isolation and characterization to populate databases with novel curated archaeal virus genomes.

In conclusion, the analysis of hot springs still holds great potential for virus (and their hosts) discovery, and a substantial number of additional springs needs to be investigated in order to derive a global ecological understanding of host-virus ecology and interactions in these unique terrestrial ecosystems.

## Figures and Tables

**Table 1:** Morphological and quantitative measurements family-level classification of observed virus-like particles (VLPs).

Virus Type/Family	No. of Particles	Virion Dimensions (in nm)	
		Nucleocapsid Diameter Range	Tail Length
*Myoviridae*	6	66–129	111–117
*Siphoviridae*	51	49–111	58–318
*Podoviridae*	5	62–70	15–41
*Fuselloviridae*-like	8	112–160 (40–99) ^1^	-
Spherical, unknown	4	66–100	-

^1^ Virion width range.

**Table 2:** Most represented bacteriophage isolate genomes in the Brandvlei metavirome.

Virus Family	No. of BLASTp Hits ^a^	Most Represented Isolates
*Myoviridae*	101	*Bacillus* virus G
*Siphoviridae*	23	Cyanophage PP
*Podoviridae*	12	*Synechococcus* phage S-SKS1
Unclassified	5	*Prochlorococcus* phage Syn1

^a^ Number of putative proteins with 30% or more similarity.

**Table 3:** Identification of putative virus hosts through interspaced short palindromic repeats (CRISPR) sequence matches.

Domain	Species Name	Sequence Accession Number	Gene Function ^1^
Bacteria	*Enterobacter sakazakii*	NC_009778	Tail tape measure
	*Luteipulveratus mongoliensis*	NZ_CP011112	Tail tubular protein
	*Capnocytophaga canimorsus*	NC_015846	Terminase, large
	*Salmonella enterica*	NZ_CP012349	Unknown
	*Thermoanaerobacterium thermosaccharolyticum*	NC_019970	Portal protein
	*Acinetobacter* sp*.*	NC_005966	Terminase, large
	*Spirochaeta caldaria*	NC_015732	Phage scaffolding protein
	*Myxococcus fulvus*	NZ_CP006003	Unknown
	*Pseudomonas* sp*.*	NZ_CP010892	Terminase, large
Archaea	*Pyrococcus* sp*.*	NC_015474	Portal protein
	*Thermococcus* sp*.*	NC_016051	Tail fiber protein
	*Pyrobaculum* sp*.*	NC_016645	Terminase, large
	*Methanothermobacter* sp*.*	NZ_AP011952	Unknown
	*Thermococcus piezophilus*	NZ_CP015520	Unknown

^1^ Gene region that contained the spacer sequence.

## Supplementary Materials

The following are available online at www.mdpi.com/1999-4915/9/11/348/s1.
