# Correction: Zablocki, O.; *et al*. Diversity of dsDNA Viruses in a South African Hot Spring Assessed by Metagenomics and Microscopy. *Viruses* 2017, *9*, 348

**DOI:** 10.3390/v10010017

**Published:** 2018-01-02

**Authors:** Olivier Zablocki, Leonardo Joaquim van Zyl, Bronwyn Kirby, Marla Trindade

**Affiliations:** Institute for Microbial Biotechnology and Metagenomics, Department of Biotechnology, University of the Western Cape, 7535 Bellville, South Africa; olivier.zablo@gmail.com (O.Z.); vanzyllj@gmail.com (L.J.v.Z.); bkirby@uwc.ac.za (B.K.)

The authors wish to make the following changes to their paper [1]. In Section 3.5.1. (Identification Using the *polB2* Gene), we erroneously grouped His1 and His2 viruses in the same genus, *Salterprovirus*, and had concluded that our metagenomic sequences which had grouped with His1 and His2 viruses had expanded the *Salterprovirus* clade (Figure 2, [1]). His1 virus is the sole current representative of the genus *Salterprovirus* [2]. However, His2 virus is classified as belonging to the genus *Gammapleolipovirus* [3]. Thus, metagenomic sequences related to both His1 and His2 viruses may be expanding either genera or both, and not only the genus *Salterprovirus*, as initially stated in the submitted paper.

The authors apologize for any inconvenience this may have caused. The changes do not significantly affect the scientific results however rectify the taxonomic inaccuracies.
