# Supplementary material for: Diversity of dsDNA Viruses in a South African Hot Spring Assessed by Metagenomics and Microscopy
Source: Viruses. 2017 Nov 18;9(11):348. doi: 10.3390/v9110348 (PMC5707555; doi:10.3390/v9110348)
Supplement: Supplementary file 1 [file viruses-09-00348-s001.pdf]

Supplementary Tables (3)

Table S1. Overall NGS run statistics, QC and metrics.

**Run/assembly/annotation**	
Post-QC no. of reads	869029
No. of contigs	9215
N50	1266 bp
Min/max contig size	200/26950 bp
Mean GC%	53.74%
No. of predicted genes	19053
No. of tRNA genes	69
No. of rRNA genes (5S, 16S, 23S)	12
No. of Pfam protein clusters	2717
**Diversity metrics**	
Richness	991
Evenness	0.964
Most abundant virotype	3.64%
Shannon-Wiener Index	6.65

Table S2. Putative functional virus genes identified in Archaea-annotated virome genes.

**Archaea phylum**	**Species**	**COG/pfam gene name hit in host genome**	**COG ID**	**% ID**
Candidatus *Micrarchaeota*	unclassified	ATP-dependent DNA ligase	COG1793	44.33
Crenarchaeota	*Pyrobaculum sp.* 1860	Superfamily II DNA or RNA helicase	COG1061	36.36
Euryarchaeota	*Ferroglobus placidus*	ERCC4-type nuclease	COG1948	34.43
		Ribonucleotide reductase alpha subunit	COG0209	51.19
	Archaeoglobus sulfaticallidus	Terminase-like family protein	pfam03237	43.79
	Haloferax denitrificans	Phosphoribosylamine-glycine ligase	COG0151	31.53
	Haloferax sp.	Uracil-DNA glycosylase/ DNA polymerase III subunits	COG1573	37.34
	Halovenus aranensis	DNA modification methylase/ DNA polymerase III subunits	COG0863	51.56
	Haloarcula sinaiiensis	Phosphoribosylamine-glycine ligase	COG0151	30.22
	Haloarcula japonica	phosphoribosylamine--glycine ligase	COG0151	42.37

	Halogranum rubrum	Predicted 5' DNA nuclease	COG3743	31.76
	Haloarcula argentinensis	Uncharacterized protein YjbI, contains pentapeptide repeats	COG1357	59.15
	Methanoregula boonei	DNA modification methylase	COG0863	75.68
	Methanothermus fervidus	peptidase u32	pfam01136	30.41
	Methanobrevibacter curvatus	Baseplate J-like protein	pfam04865	37.11
	Methanoplanus petrolearius	DNA modification methylase	COG0863	31.14
	Methanolobus psychrophilus	Large terminase	COG5323	58.98
	Methanococcoides methylutens	2-polyprenyl-3-methyl-5-hydroxy-6-metoxy-1,4-benzoquinol methylase	COG2227	35.84
	Methanomethylovorans hollandica	Phage terminase large subunit	COG5362	35.42
	Methanosarcina mazei	Recombination protein Bet	TIGR01913	39.74
	Methanoculleus sp. MAB1	DNA modification methylase	COG0863	47.54
	Methanosaeta thermophila	Adenylate kinase	COG0563	46
	Methanofollis ethanolicus	Prephenate dehydrogenase	COG0287	40.49
	Methanosarcina sp. E03.2	CRISPR/Cas system-associated endoribonuclease Cas2	COG1343	42.86
	Thermococcus onnurineus	Hypoxanthine phosphoribosyltransferase	COG2236	31.21
	Methanogenic archaeon ISO4-H5	Deoxycytidylate deaminase	COG2131	50.91
Thaumarchaeota	*Candidatus Nitrosocosmicus*	DNA modification methylase/pol 3 subunit	COG0863	65
	Nitrosopumilus maritimus	2-alkenal reductase	GO0032440	36.13
	Candidatus Nitrosopumilus sp. AR2	DNA topoisomerase-1	COG3569	38.36
	Thaumarchaeota archaeon MY3	DNA modification methylase	COG0863	65

Unclassified	*Halobacteriaceae archaeon* HSR2	DNA polymerase elongation subunit (family B)	COG0417	37.63

Table S3. Reference assembly trials to all RefSeq Archaeal genomes at the nucleotide level using CLC Genomics.

**Length/ similarity fraction %**	**Number of reads with a hit**	**Best archaeal virus genome match**
0.5/0.5	626089	HCTV-1
0.5/0.6	217543	HCTV-2
0.5/0.7	7351	HCTV-2
0.5/0.8	1444	HCTV-2
0.6/0.5	624805	HCTV-1
0.6/0.8	792	*Sulfolobus* virus STSV2
0.7/0.5	623059	HCTV-1
0.7/0.8	474	*Sulfolobus* virus STSV2
0.8/0.5	620669	HCTV-1
0.8/0.8	283	*Sulfolobus islandicus* rudivirus 3
0.95/0.65	2565	HCTV2

Supplementary Figures (3)

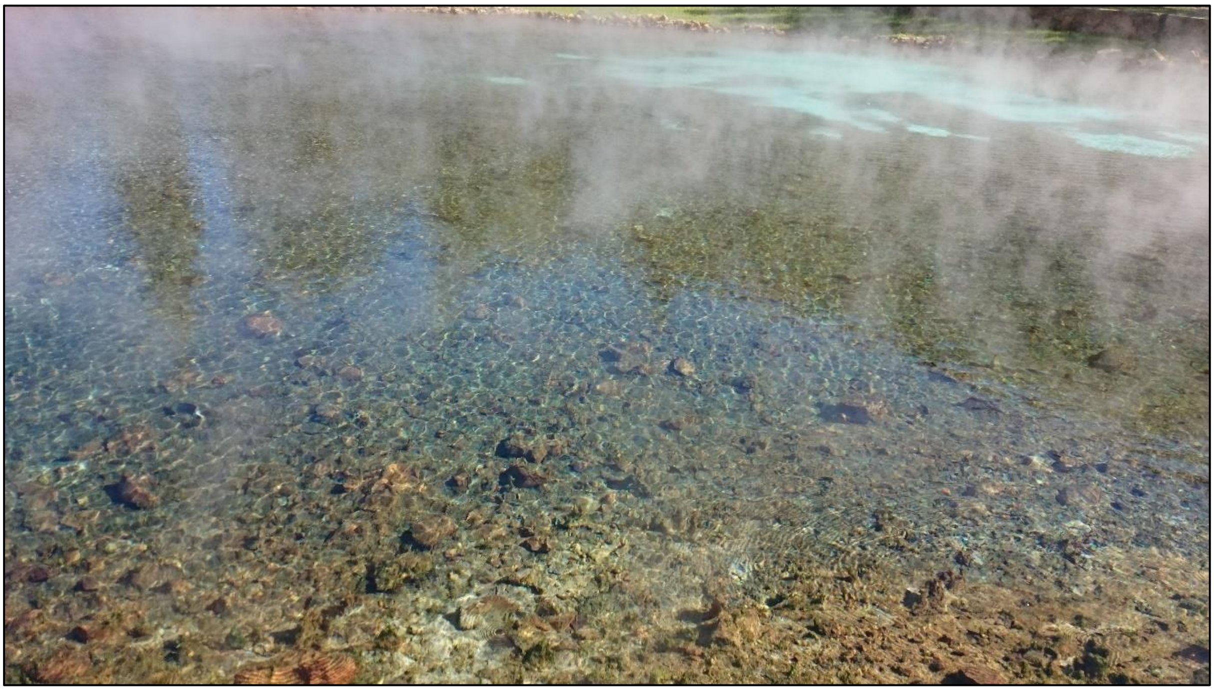

Figure S1. The sampling site used in this study, Brandvlei hot spring.

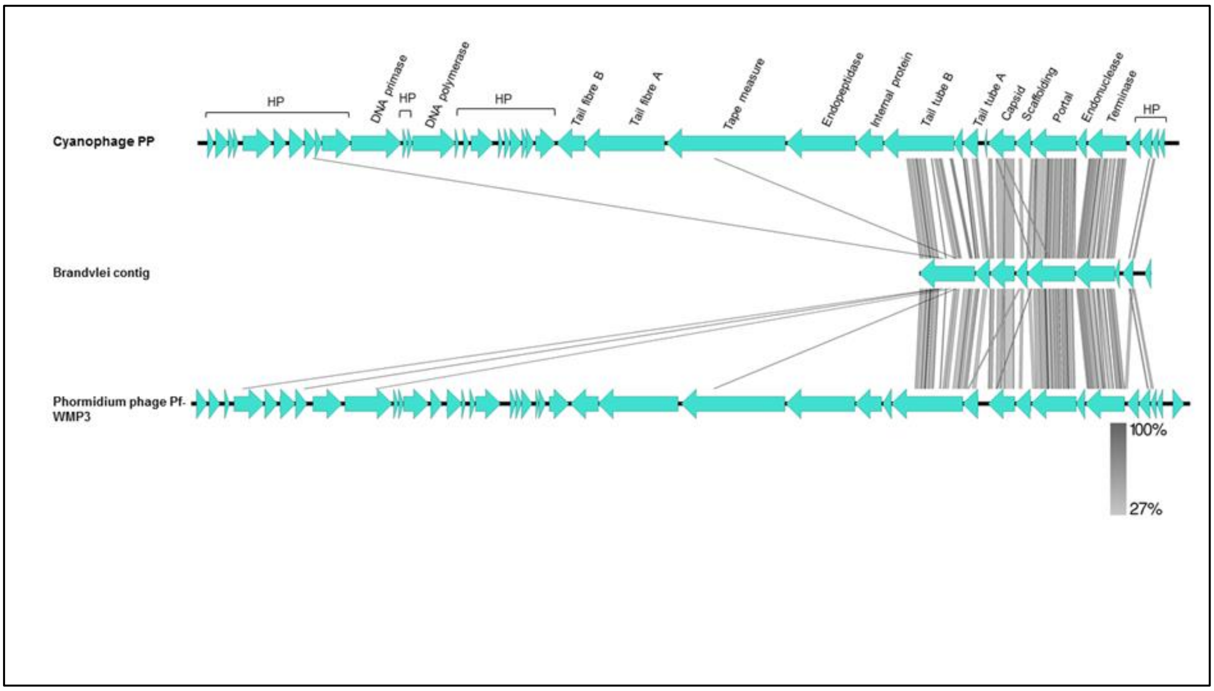

Figure S2: Full genome comparison (using tBLASTx) between reference cyanopodoviruses genomes (Cyanophage PP and *Phormidium* phage) and the most abundant Brandvlei contig (192 × coverage). Drawn with EasyFig 2.2.2.

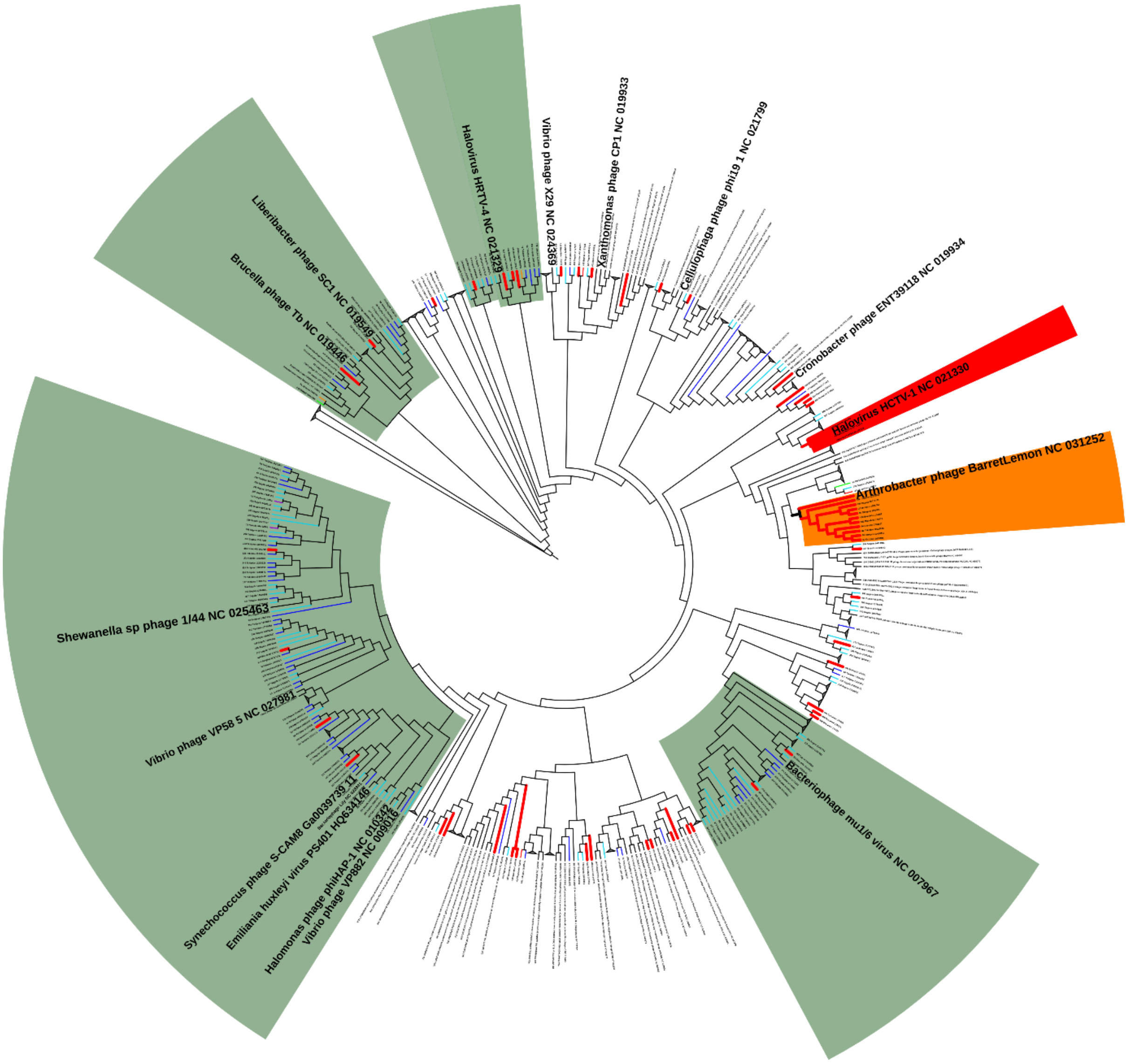

Figure S3. Phage diversity in Brandvlei hot spring using the large terminase (terL) amino acid sequence. For reading clarity, branch length was turned off, and clades containing sequences with no close homology to hot spring sequences were collapsed and are depicted by inverted grey triangles. The UPGMA method was used to infer genetic relatedness using the full-length amino acid sequence of the terminase gene (see materials and methods). Green shadings represent phage clusters composed of phages mostly isolated from a hot spring source. Individual branch colourings indicate phage terminase exclusively associated with hot spring phages, including Brandvlei (red), Tshipise (dark blue), Sagole (light blue), Great Boiling Spring (light green), and Octopus Spring (orange). In addition, orange shading indicates a cluster of *Arthrobacter* phage-like sequences and the red cluster indicates Halovirus-like phages. The tree was drawn and edited with iTOL.
